# Conformational Dynamics of a Ligand-Free Adenylate Kinase

**DOI:** 10.1371/journal.pone.0068023

**Published:** 2013-07-05

**Authors:** Hyun Deok Song, Fangqiang Zhu

**Affiliations:** Department of Physics, Indiana University-Purdue University Indianapolis, Indianapolis, Indiana, United States of America; Oak Ridge National Laboratory, United States of America

## Abstract

Adenylate kinase (AdK) is a phosphoryl-transfer enzyme with important physiological functions. Based on a ligand-free open structure and a ligand-bound closed structure solved by crystallography, here we use molecular dynamics simulations to examine the stability and dynamics of AdK conformations in the absence of ligands. We first perform multiple simulations starting from the open or the closed structure, and observe their free evolutions during a simulation time of 100 or 200 nanoseconds. In all seven simulations starting from the open structure, AdK remained stable near the initial conformation. The eight simulations initiated from the closed structure, in contrast, exhibited large variation in the subsequent evolutions, with most (seven) undergoing large-scale spontaneous conformational changes and approaching or reaching the open state. To characterize the thermodynamics of the transition, we propose and apply a new sampling method that employs a series of restrained simulations to calculate a one-dimensional free energy along a curved pathway in the high-dimensional conformational space. Our calculated free energy profile features a single minimum at the open conformation, and indicates that the closed state, with a high (∼13 kcal/mol) free energy, is not metastable, consistent with the observed behaviors of the unrestrained simulations. Collectively, our simulations suggest that it is energetically unfavorable for the ligand-free AdK to access the closed conformation, and imply that ligand binding may precede the closure of the enzyme.

## Introduction

Adenylate Kinase (AdK) is a small ubiquitous enzyme that catalyzes the reversible phosphoryl-transfer reaction 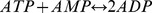 and plays an important role in cell signaling [1], [2] and energy metabolism [1]. Malfunction of AdKs may cause human diseases such as nucleotide diphosphate kinase deficiency [3], hematopoietic defect [4], and hemolytic anemia [5]. Similar to many enzymes, the catalytic cycle of AdK involves large-scale conformational change of the protein. Indeed, AdK structures in different conformational states have been captured in crystallography and NMR experiments [6]–[12]. In particular, the ligand-free AdK conformation appears to be in an open state [7], in contrast to the nucleotide-bound crystal structure in an apparently closed state [6]. Structurally, AdK is composed of three domains: an ATP-binding domain (LID: residues 122–159), an AMP-binding domain (AMPbd: residues 30–59), and the CORE domain (residues 1–29, 60–121, and 160–214) [13], [14]. The relative positions and orientations of the AMPbd and LID domains characterize the major difference between various AdK conformations.

Apart from experimental investigations [10], [15], [16], conformational transitions in AdK have also been extensively studied in molecular simulations [13], [14], [17]–[20] employing a variety of sampling techniques. Many simulation studies focus on the ligand-free state of AdK, which is believed to be part of the catalytic cycle. To name a few, Kubitzki and de Groot identified a transition pathway between the open and the closed conformations of ligand-free AdK, using temperature-enhanced essential dynamics replica exchange [13]. Arora and Brooks [17] computed the free energy as a function of the difference in the root mean square deviations (RMSDs) with respect to the open and closed crystal structures. Beckstein et al. applied dynamic importance sampling to reveal the conformational changes [14]. More recently, using the string method [21], Matsunaga et al. calculated the free energy profiles along the transition pathways for the ligand-free and ligand-bound AdK [18].

Currently, simulations employing different sampling methods do not seem to have reached a consensus conclusion concerning the AdK conformations. For the ligand-free AdK, e.g., some calculated free energies indicate that the closed conformation would not be stable, whereas other studies suggest that it is a metastable state instead. In this study, we aim to examine the stability and dynamics of the open and closed AdK conformations using molecular dynamics simulations. Specifically, our simulations are designed to offer insight into the following questions: Which conformation would a ligand-free AdK predominantly adopt at equilibrium? Are both the open and closed conformations metastable? What is the difference in the equilibrium probability (or equivalently, the free energy) between the two conformations?

To help answer the questions above, we carry out two types of simulations here. The first type involves simulations starting from the open or the closed conformation of a ligand-free AdK, without any applied restraints. These unrestrained simulations could offer a robust and unbiased test on the stability of a given protein conformation, as they are not subject to the assumptions and approximations involved in the various enhanced sampling methods. Indeed, unrestrained simulations of AdK were reported in several earlier studies [13], [22], [23]. Brokaw and Chu simulated the open and closed conformations of AdK with and without the bound ligand [22], and observed some complete or partial spontaneous transitions between the two conformations. Ramanathan et al. also performed unrestrained simulations starting from the two AdK conformations, and analyzed the trajectories using a novel quasi-anharmonic technique [23]. Currently, the outcomes from the unrestrained simulations appear to vary somewhat from study to study. For the closed-state ligand-free AdK, e.g., in some simulations a complete closed-to-open transition was observed within ∼100 ns, whereas in others only a partial opening event occurred. Such variation could arise either from the differences in the simulation protocols (protein force field, water model, etc.), or from the intrinsic protein flexibility. To clarify this issue, here we initiate multiple unrestrained simulations from each state and observe the subsequent free evolutions of the protein conformation. The multiple simulations using the same protocol and initial structure can thus reveal the intrinsic diversity in the conformational dynamics of AdK.

Although the unrestrained simulations above may provide valuable information on the stability of the given conformations, the currently affordable simulation time is orders-of-magnitude shorter than what is needed to fully sample the conformational space. If two metastable conformations are separated by some energetic barrier, it would be very unlikely to observe, even in multiple simulations, any spontaneous transition. Therefore, to complement the unrestrained simulations, we also carry out another type of calculation here, in which we employ a series of biased simulations to estimate the free energy in the conformational space.

In general, protein conformations require high-dimensional representations, and are described by a relatively large number of chosen “coarse coordinates”, which can be either Cartesian coordinates [24] or collective variables [21]. These coarse coordinates define a high-dimensional configuration space, and each point in this space represents a conformation. A free energy as a function of the coarse coordinates can be defined by integrating out all other (such as solvent) degrees of freedom. The objective of the conventional string method is then to identify a “minimum-free-energy-pathway” [21] that connects two free energy minima in the configuration space, each representing a metastable conformational state. However, although the configuration space represents a significant dimensionality reduction compared to the entire phase space, it is still of relatively high dimensions. The multidimensional free energy associated with this pathway, as obtained from the conventional string method, only describes the energetics on a single curve, and ignores information along the directions perpendicular to the curve. This is apparently not desired, given the high dimensionality of the configuration space. One approach to alleviate this problem is to adopt a low-dimensional configuration space, e.g., by using only a small number of linear modes from a principal component analysis to describe the protein conformation [18]. Alternatively, transitions between two conformations can be described by transition (or reaction) tubes [25], [26] in the high-dimensional configuration space, as further discussed below.

A transition tube [25], [26] refers to a region in the configuration (conformational) space that connects the two metastable conformational states, such that most spontaneous transitions between the two states go through this tube. The center of the transition tube is defined as its principal curve [25], and a free energy can be defined along this curve. Unlike the multidimensional free energy associated with the minimum-free-energy-pathway discussed earlier, however, this free energy has further integrated out all degrees of freedom perpendicular to the principal curve, and is thus a one-dimensional function of the curve parameter alone. Although the transition is now described by a single progression (curve) parameter, we note that the principal curve still lies in a high-dimensional configuration space, and the curve parameter is essentially a collective variable based on all coarse coordinates. This approach thus minimizes the possibility of ignoring important degrees of freedom from the description. It was recently demonstrated [26], [27] that the one-dimensional free energy associated with the principal curve represents a significant advancement over the minimum-free-energy-pathway in the conventional string method.

The one-dimensional free energy can be calculated from confined or restrained simulations. A recent approach involves sampling in the Voronoi tessellation with reflective boundaries [26], [27]. Here we present an alternative approach. By invoking a local linear approximation, we employ traditional one-dimensional umbrella sampling to calculate the free energy along the principal curve. Our method is conceptually simple, computationally efficient, and relatively convenient to implement. We thus use this technique to map a free energy profile in the conformational space visited by the unrestrained simulations, thereby supplementing the free conformational dynamics of AdK with the thermodynamic energetics.

## Methods

### Unrestrained Simulations

**Figure 1:**
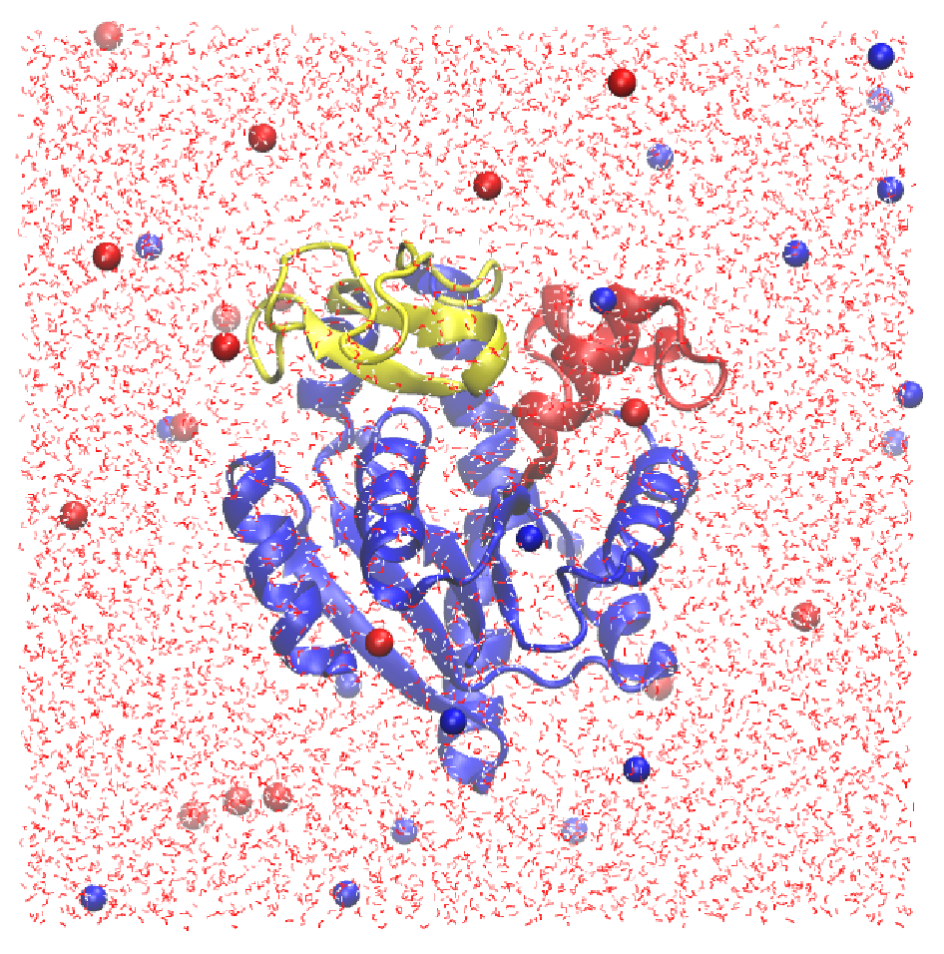
The simulation system. AdK was initially in the closed conformation in this particular simulation. The AMPbd, LID, and CORE domains of the protein are colored red, yellow, and blue, respectively. K+ and Cl^−^ ions are drawn as blue and red spheres, respectively. The image was rendered using the VMD software [45].

We constructed two simulation systems with AdK initially in the open and closed conformations, respectively. The atomic coordinates of the protein were taken from monomer A in the crystal structures (PDB ID: 4AKE [7] and 1AKE [6] with the bound nucleotide analog removed) for the open and closed states, respectively. We adopted the standard protonation states at pH 7 for all residues. In particular, all His residues are neutral, with the proton at the 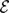 position. For both systems, the protein was solvated by adding 8,900 water molecules in a cubic box. 21 K^+^ and 17 Cl^−^ were also added to each system, mimicking a KCl concentration of ∼0.1 M. In both cases the simulation system (Fig. 1) contains a total of 30,079 atoms.

For each system, we first fixed the entire protein and equilibrated the water and ions for 1 ns. Next, we relaxed the protein and applied harmonic restraints on the C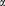 atoms only, and further equilibrated the system for 2 ns. We then selected seven and eight snapshots from the open- and closed-state simulation trajectories above, respectively. Starting from the selected snapshots in the open-state trajectory, we performed seven simulations (O1–O7), in which the entire system is subject to no restraint and is free to evolve. Similarly, we initiated eight unrestrained simulations (C1–C8) from the closed state. The fifteen unrestrained simulations were each run for 100 ns, with four of them extended to 200 ns, as will be described in Results.

All simulations were performed using the CHARMM (Ver. c36) protein force field [28]–[30], the TIP3P water model [31], and the NAMD2 (Ver. 2.9) program [32], with a time step of 2 fs. All bond lengths involving hydrogen atoms were constrained using the SHAKE [33] and SETTLE [34] algorithms. We adopted a cutoff distance of 12 Å for nonbonded interactions, with a smooth switching function taking effect at 10 Å. Full electrostatics was calculated every 4 fs using the particle-mesh Ewald method [35]. Temperature was maintained at 300 K by Langevin dynamics with a damping coefficient of 0.1 ps^−1^. A constant pressure of 1 atm was achieved using the Nose-Hoover Langevin piston method [36], with the volume of the periodic box allowed to fluctuate but the cubic geometry strictly fixed. During the equilibration, the length of the periodic box was stabilized at ∼67 Å.

### Free Energy Sampling

To further explore the thermodynamics of the conformational space, we first obtained a conformational pathway from the trajectories of the unrestrained simulations, as described below, and then carried out a set of restrained simulations to calculate the free energy profile along this pathway. Throughout this study, the protein conformation is represented by the positions of its *N* = 214 C*_α_* atoms, or the 3*N* = 642 Cartesian coordinates. Any particular conformation *i* is thus denoted by a vector 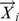 in this 3*N*-dimensional space.

Applying the concept of principal curve [25], we obtained a pathway that represents the conformational space visited in the unrestrained simulations. Specifically, we first defined a straight line, in the 3*N*-dimensional space, that connects the open and closed AdK crystal structures, 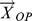 and 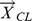, after proper alignment. For each frame *i* in the trajectories of the unrestrained simulations, we performed a rigid-body alignment with respect to the open-state crystal structure 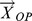, thus removing the overall translation and rotation of the protein. The protein coordinate after the alignment, 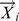, was then orthogonally projected onto the line 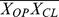 above, with the projected point denoted by 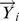. The entire range covered by all the 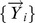 on the line was then evenly divided into 100 segments. The entire set of coordinates 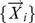 was thus also classified into 100 groups, according to the line segment each corresponding 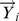 lies in. Then for each group *k*, we calculated the average coordinate, 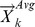, over all the coordinates in the group. These average coordinates 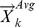 (*k* = 0,…,99) thus delineate a curve that lies at the center of the conformational space visited by the protein.

To obtain a smooth pathway through the average coordinates above, we applied multidimensional curve fitting [24], [37]. The fitted curve is of the form 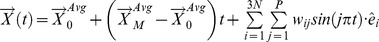, with *M* = 99 and 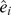 the axis for the *i*-th dimension [24]. In this study we adopted *P* = 1 such that the fitted curve has a relatively small curvature. We minimized the objective function 
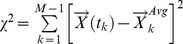
 through a multidimensional nonlinear optimization with respect to both 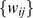 and 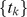 [24]. With the obtained 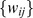, 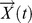 thus defines a continuous curve from 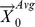 to 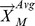, as the curve parameter *t* varies from 0 to 1. We use 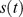 to denote the arc length [24] between 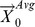 and 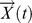 along this curve, thus with 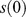 = 0, and 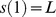 denoting the entire length of the curve between 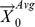 and 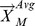 in the 3*N*-dimensional space. In general, 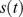 is not a linear function of *t*. We may, however, define a new curve parameter 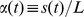. Because 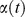 is a monotonic function, the inverse function 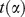 is well defined, with each 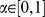 corresponding to a unique 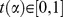, and thus a unique conformation 
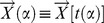
 on the curve. In this new parametrization, the arc length of the curve is a linear function of 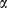. We have thus obtained a uniformly parametrized smooth pathway 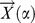, 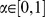, that represents the sampled conformational space in the unrestrained simulations. We note that a curve can also be obtained by simple piece-wise linear or spline interpolation after applying some smoothing techniques [21]. In this particular study, we chose to adopt the global sinusoidal representation above because the resulting curve is infinitely differentiable everywhere, thus suitable for the local linear approximation discussed below.

Any conformation near the curve 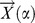 above can be projected onto the curve. Specifically, we define a projection function 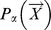, with the function value 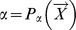 corresponding to the 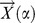 on the curve with the shortest distance to 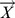. In this way we can project the entire accessible conformational space of the protein onto this one-dimensional curve in the 3*N*-dimensional coordinate space. A free energy, 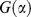, can accordingly be defined as a function of the curve parameter 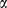 [25]–[27].

We carried out a set of umbrella-sampling simulations to compute the free energy profile 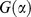, with a total of 30 umbrella windows. Each window *i* samples the vicinity of a corresponding reference 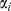. These references 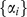 (*i* = 1, …, 30) cover the range of [0,1], with a uniform spacing of 1/29. We adopted harmonic umbrella potentials 
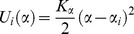
 for each window. In the coordinate space, this potential is of the form.
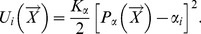
(1)


In principle, the projection 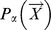 above is a nonlinear function. However, in the close vicinity of 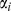, the curve can be approximated by a straight line:
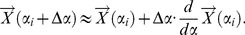
(2)


As described earlier, by construction the arc length of 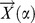 is a linear function of 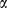, such that the magnitude of the derivative, 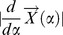, is a constant along the curve. Because the total curve length is *L* between 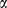 = 0 and 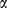 = 1, we further have 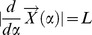, and thus can rewrite Eq. 2 as
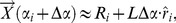
(3)in which 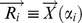, and 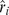 is the unit vector along the direction of 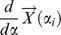, or the tangent of the curve at 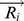. With this approximation, in the space near 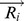, 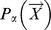 becomes a linear projection: 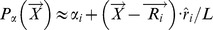. The umbrella potential (Eq. 1) thus becomes
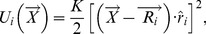
(4)in which 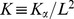. The resulting forces on the Cα atoms are then given by



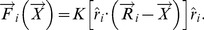
(5)Our curve 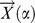 (in the 642-dimensional space) has a length of *L* = 197.2 Å between 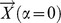 and 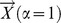. We adopted *K* = 0.08 kcal/mol/Å^2^, or 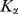 = 3,111 kcal/mol in these restrained simulations. As described above, the reference position 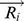 and the tangent vector 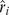 for each umbrella window were taken from the curve 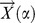 at the corresponding 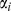.

In comparison to the multidimensional restraining potentials commonly used in the conventional string method [21], [24], our potential (Eq. 4) here only affects motions along the pathway (curve) direction, with all other degrees of freedom unrestrained. The potential only depends on the projection of the given conformation on the pathway, and the restraining force (Eq. 5) is along the pathway tangent. Consequently, the reconstructed 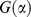 is also a one-dimensional free energy profile, with all the perpendicular degrees of freedom essentially integrated out. It was recently formulated and demonstrated that, compared to the high-dimensional free energy profiles, such one-dimensional function is a better characterization of the transition tube, and is expected to be less sensitive to the particular choice of the representative coarse coordinates [27].

We note that our restraining potential, as a function of the Cartesian coordinates, is not guaranteed to be invariant upon a rigid-body translation or rotation of the entire protein. To eliminate such effects, we applied additional restraints in all umbrella-sampling simulations. Specifically, a harmonic restraint, with spring constant of 1,000 kcal/mol/Å^2^, was applied on the center of the protein. In addition, using the crystal structure 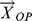 as the reference, we applied another harmonic restraint, with spring constant of 200 kcal/mol/degree^2^, on the orientation angle of the protein. These restraints eliminated the drift along the six degrees of freedom for rigid-body translation/rotation, such that the Cartesian coordinates 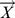 represent only the internal degrees of freedom, or the conformational state of the protein.

To enhance the sampling, we implemented Hamiltonian replica exchange [38] in the umbrella-sampling simulations. At every 400 fs, two adjacent simulations *i* and *j* attempt to swap their restraining potentials, which would result in a change in the combined Hamiltonian by 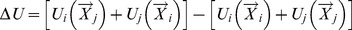. The attempt is accepted if 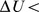 0, or otherwise accepted with a probability of exp 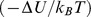. Each umbrella-sampling simulation was run for 40 ns, with the last 30 ns used for analysis. The free energy 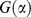 was computed using the weighted histogram analysis method [39], [40], with the statistical errors estimated from the uncertainties in the average sampled coordinate [40].

## Results

In this section, we first describe the results of our unrestrained simulations, in which some spontaneous transitions from the closed to the open conformation were observed. We then present the free energy profile along a transition pathway, as calculated from our biased sampling simulations.

### Spontaneous Conformational Transitions

As described in Methods, we performed eight unrestrained simulations (C1–C8) starting from the closed-state crystal structure, after removing the bound ATP analog. In four (labeled C1–C4) of these simulations (Fig. 1B), AdK immediately underwent a fast spontaneous transition toward the open conformation within the first ∼5 ns. Following this initial phase, the protein exhibited substantial conformational fluctuations, but appeared to be stabilized in conformations near the open crystal structure (Fig. 1, green dashed lines) by the end of the simulations. In the other four simulations (labeled C5–C8), in contrast, AdK did not complete the transition within the 100 ns simulation time. We thus extended C5–C8 by another 100 ns. During the extended simulation time, in one simulation (C5) the protein made a sharp transition and reached the open state (Fig. 1C). The protein also approached the open state in two other simulations (C6 and C7), although apparently not having fully completed the transition (Fig. 1C). In the other simulation (C8), in contrast, the protein stayed near the closed state during the entire simulation time (Fig. 1C).

**Figure 2:**
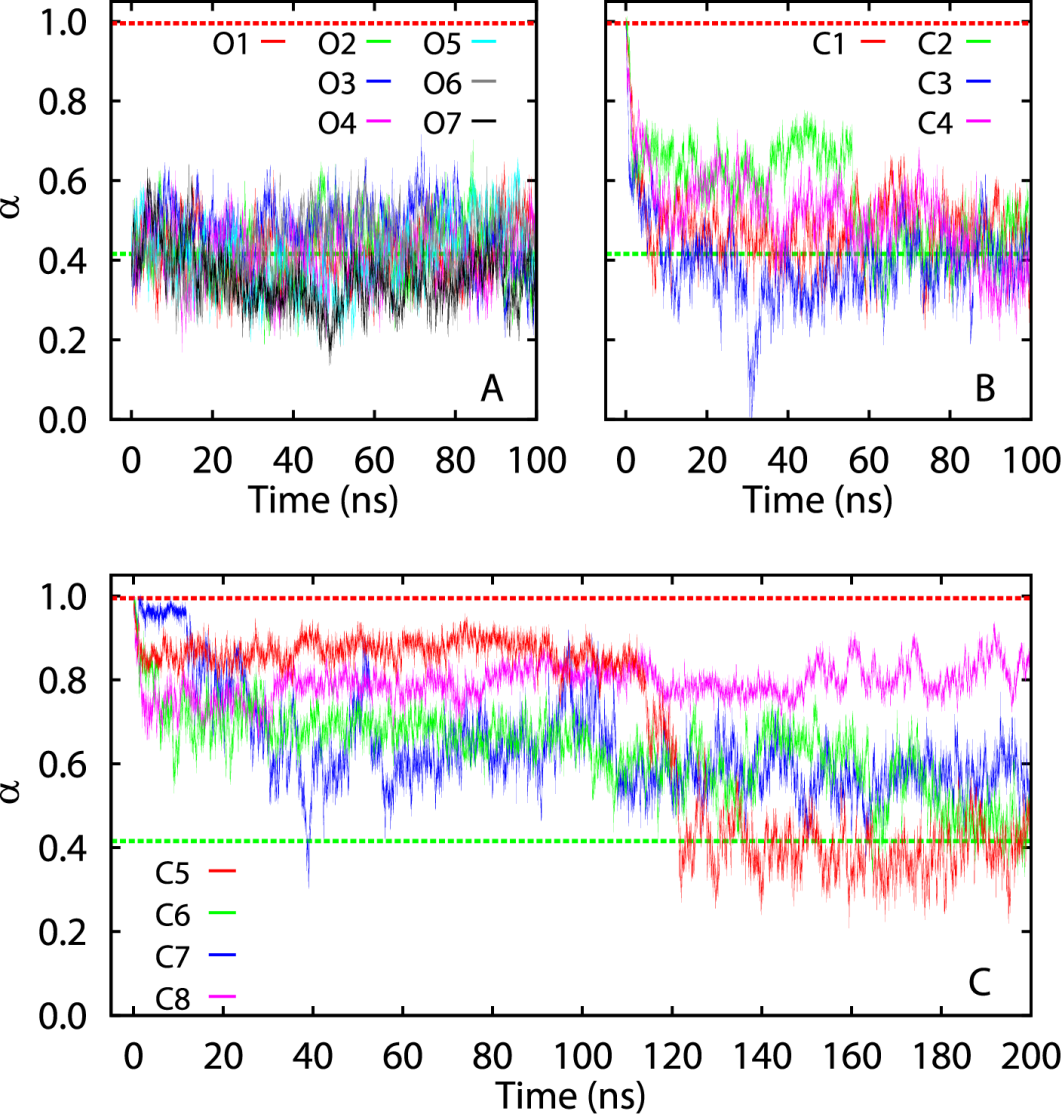
Time evolution of the unrestrained simulations along a conformational pathway. The pathway is represented by a parameterized curve 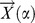 (see Methods). Each protein conformation 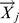 in the simulation trajectories was projected onto this curve through the operator 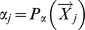 (see Methods), which returns the curve parameter 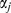 corresponding to the point 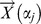 on the curve with the shortest distance to 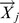. This operator was implemented through a nonlinear minimization algorithm. The projected curve parameter is plotted as a function of time for each simulation trajectory. The green and red dashed lines indicate the projected curve parameters for the open (*α* = 0.42) and closed (*α* = 0.99) crystal structures, respectively.

We also carried out seven unrestrained simulations (O1–O7) starting from the open-state crystal structure. Similar to C1–C8, in these simulations considerable conformational fluctuations of the protein were observed (Fig. 2A). However, in all simulations (O1–O7) AdK remained in the vicinity of its initial state and never visited the conformational space near the closed state (Fig. 2, red dashed lines).

For each of the simulations above (C1–C8, O1–O7), we averaged the C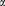 coordinates over the frames in the last 40 ns of the trajectory, after proper alignment. These average coordinates thus represent the protein conformations in the latest stage of the simulation. Table 1 compares the average conformation from each simulation to the two crystal structures. The RMSD values clearly indicate that in all simulations except C8, regardless of the initial state, the protein eventually adopted conformations much more similar to the open-state crystal structure than to the closed state. These unrestrained simulations (Fig. 2 and Table 1) thus suggest that for the ligand-free AdK, the open state represents the favored conformation, as all observed transitions were in the closed-to-open direction. Similar behaviors were also observed in previous unrestrained simulations [13], [22].

**Table 1:** RMSDs between the average protein conformations from the unrestrained simulations and the AdK crystal structures.

	C1	C2	C3	C4	C5	C6	C7	C8	O1	O2	O3	O4	O5	O6	O7
Open (Å)	2.19	1.38	1.99	1.79	1.21	2.01	3.06	6.35	1.64	1.03	1.51	1.15	1.57	1.61	1.55
Closed (Å)	6.53	7.00	7.38	6.80	7.63	6.04	5.40	3.26	7.16	6.85	6.82	7.23	6.78	6.76	8.22

The protein conformation (represented by its C*_α_* coordinates) from each frame in the simulation trajectories was aligned against the crystal structure, and the mean conformation for each simulation was obtained by averaging the aligned C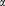 coordinates over the frames in the last 40 ns of the simulation. These mean conformations were compared to the open (4AKE) [7] and the closed (1AKE) [6] AdK crystal structures, with the RMSDs provided in the table.

**Figure 3:**
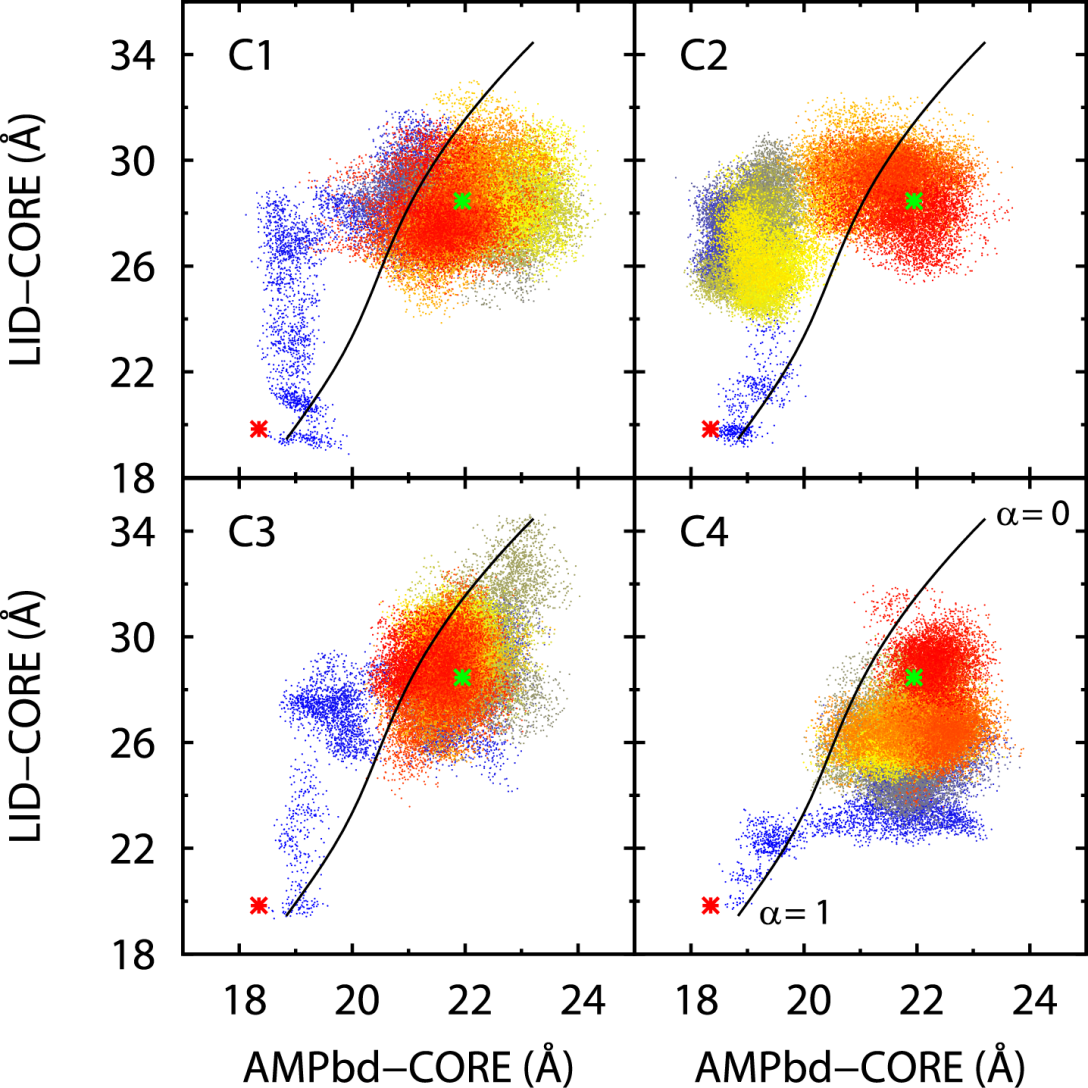
Evolution of the distances between the domain centers. The center of each (CORE, AMPbd, or LID) domain is defined by the average position of its C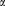 atoms. Distances between these centers are calculated for four 100-ns unrestrained simulations (C1–C4). Each frame in the simulation trajectories corresponds to one point in the figure, with the color denoting the progression of the simulation, from blue at the onset (via yellow) to red at the end of the simulation. The black curve represents a pathway averaged from all unrestrained simulations (see Methods) and used as the principal curve in the umbrella-sampling simulations. The green and red stars indicate the positions of the open and closed crystal structures, respectively.

The spontaneous transitions in simulations C1–C4 allowed us to observe the AdK conformational changes in details. Rigorous quantitative methods, such as principal component analysis [41], are available to visualize or compare the trajectories in lower dimensions. Alternatively, some simpler intuitive measures, such as the distances from the CORE domain to the AMPbd and LID domains, have been used in many studies as a reduced representation of the AdK conformation [22], [42]–[44]. Here we thus calculated the distances (Fig. 3) between the centers of the C*_α_* atoms in these domains throughout the trajectories of C1–C4. Figure 2 shows a larger change (∼10 Å) in the LID-CORE distance than that (< 5 Å) in the AMPbd-CORE distance, indicating a larger movement of the LID domain during the transition. As mentioned earlier (Fig. 2B), the transitions took place at the very beginning of simulations C1–C4, as indicated by the color code in Fig. 3. However, the exact pathway and evolution of the transitions are not identical among C1–C4. In C2, e.g., the protein stayed in some intermediate states for a substantial amount of time before reaching the final open conformation. Near the stabilized open state, the AdK conformations in all simulations occupy a substantial area in the reduced 2D graph (Fig. 3), reflecting the large conformational fluctuations. In simulation C5, a closed-to-open transition occurred at ∼120 ns (Fig. 2C), through a pathway (not shown) similar to that in C4.

**Figure 4:**
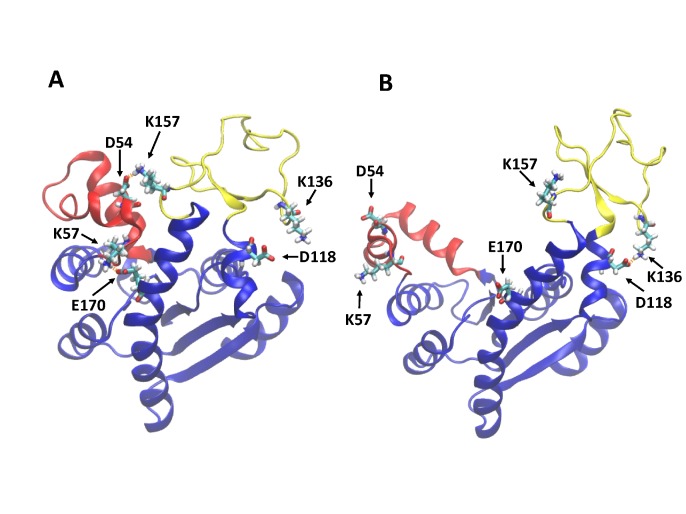
Some typical salt bridges in the closed (A) and open (B) AdK conformations. The two snapshots were taken from simulations C5 and O1, respectively. The images were rendered using the VMD software [45].

A number of charged residues are located on the AdK surface, with their side chains potentially forming salt bridges. When AdK adopted the closed conformation (as initially in C1–C8), salt bridges K57-E170 and K157-D54 were frequently observed (Fig. 4A), which link the AMPbd domain to the CORE and LID domains, respectively. In some simulations, D54 occasionally formed a salt bridge with the neighboring R156 instead of K157. These salt bridges were never present in simulations O1–O7, and were broken as the protein deviated from the closed conformation in simulations C1–C7, although K57-E170 remained for ∼50 ns in simulation C2 when AdK was in the intermediate states (Fig. 2B and Fig. 3B). In contrast, the open conformation features a stable salt bridge, K136-D118 (Fig. 4B), between the LID and the CORE domains, as highlighted in previous studies [13], [14]. This salt bridge was present in O1–O7 during the entire simulation time, and was formed in C1–C8 a few ns after the start of the simulations. In C8, the only simulation that did not significantly deviate from the closed conformation, K136-D118 was maintained in the first ∼50 ns but was then broken and not formed again, whereas the salt bridges K57-E170 and K157/R156-D54 mentioned earlier were frequently observed throughout the entire simulation. In addition, C8 features another salt bridge, R36-D158, which is not found in all other simulations. We note that whereas different criteria can be used to define salt bridges, in our description here a salt bridge is assigned only if a highly directional and specific hydrogen bond is present between the two side chains. Overall, as discussed above and shown in Fig. 4, the open AdK conformation is stabilized by the salt bridge K136-D118 [13], [14], and the closed conformation appears to favor the formation of K57-E170 and K157/R156-D54.

### Energetics of the Transition

To elucidate the conformational energetics of AdK, we applied a novel umbrella-sampling technique (see Methods) to calculate the one-dimensional free energy profile (or PMF) along a transition pathway averaged from the trajectories of the unrestrained simulations (Methods). As shown in Fig. 3 (black curve), the average pathway can also be projected onto the two inter-domain distances. Because the transitions in the individual unrestrained simulations are different, some sections of this average pathway are actually not frequently visited in those simulations. We note, however, that in our sampling simulations, the restraint only acts along the curve direction (see Methods), and does not force the conformation perpendicularly towards the curve. In each restrained simulation, the protein conformation is thus free to explore the dimensions orthogonal to the curve, and does not necessarily settle on the curve itself. We also note that when properly sampled, the free energy difference between two states should not depend on the specific pathway (route) through which the free energy is integrated.

**Figure 5:**
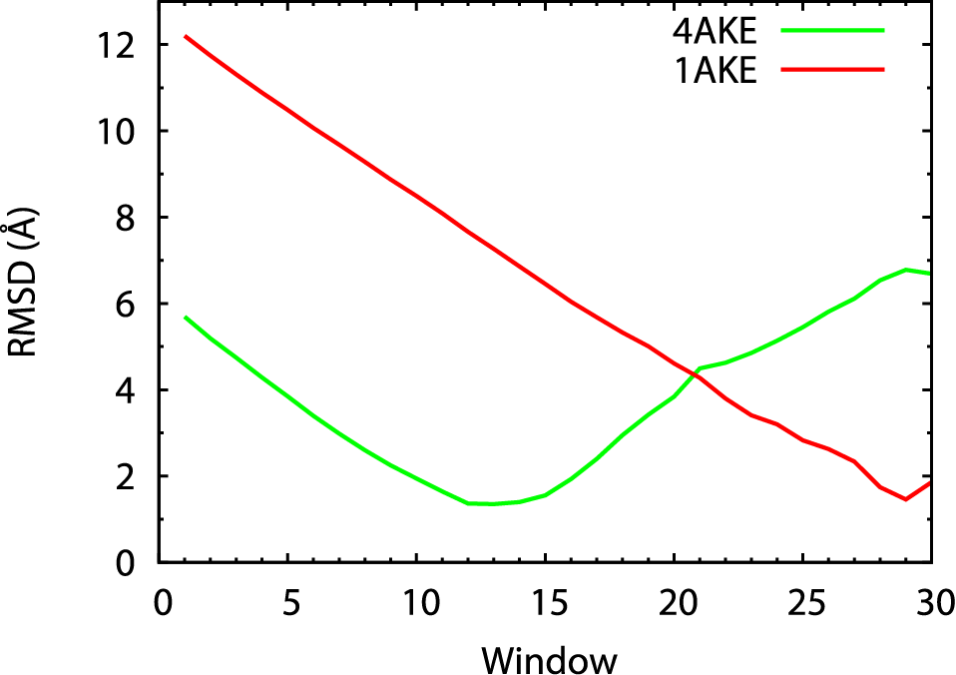
A comparison of the average conformations from the umbrella-sampling simulations to the crystal structures. Average C*_α_* coordinates for each of the 30 umbrella windows were calculated from the trajectories. The RMSDs between these average C*_α_* coordinates and the two crystal structures are plotted in the figure.

To examine the sampled conformations in the restrained simulations, we calculate the average C*_α_* coordinates from the trajectory in each umbrella window, and compared them with the crystal structures of AdK. The RMSD values in Fig. 5 indicate that conformations near the two crystal structures have indeed been sampled in the expected umbrella windows (with small RMSDs). Moreover, because the unrestrained simulations visited a large conformational space, our pathway curve extends beyond the crystal structures, especially at the open-state end (Figs. 3 and 5).

**Figure 6:**
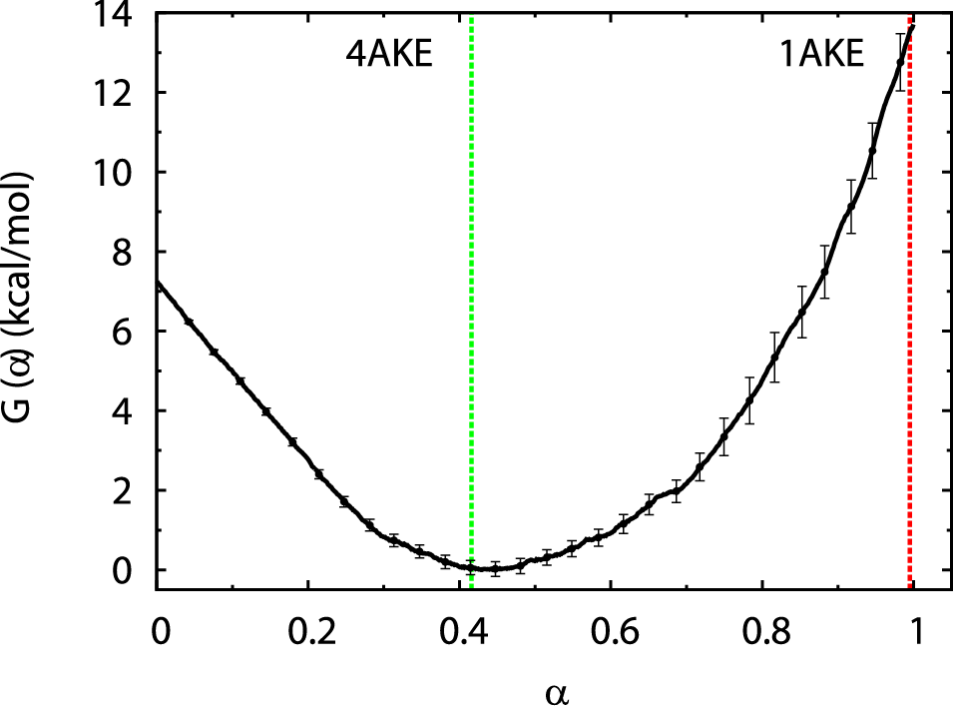
Free energy profile of AdK conformations. The free energy 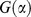 was defined along a conformational pathway (see Methods), and calculated from the umbrella-sampling simulations. The plotted error bars are for the free energy difference with respect to the first umbrella window at *α* = 0, estimated from the statistical uncertainties in the mean coordinate [40]. The green and red dashed lines indicate the projected locations of the open and closed crystal structures, respectively.

As shown in Fig. 6, the free energy overall represents a valley with a single minimum. The location of the energetic minimum, with a reduced curve parameter *α* ∼ 0.43, is almost exactly at the open-state crystal structure (*α*∼ 0.42, green dashed line), and agrees well with the sampled conformations (Fig. 2A) in the unrestrained simulations. Remarkably, with the bound ATP analog removed, the closed-state crystal structure (*α* ∼ 0.99, red dashed line) no longer represents a metastable state, as it is not in a local free energy minimum. The monotonic energy landscape there is consistent with our finding that all unrestrained simulations (Fig. 2, B and C) initiated from the closed state drifted away from that state and moved to varying extents toward the open state. As shown in Fig. 5, the free energy at the closed state is ∼13 kcal/mol above the energetic minimum. This large free energy is consistent with the fact that no unrestrained simulation initiated from the open state ever approached the closed state.

## Discussion

In this study, we applied the concepts in the finite-temperature string method [25], [26] to characterize the conformational free energy of AdK. By mapping the protein conformations onto a single parameterized curve, we define a free energy 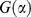 along this curve. Although the protein conformation is still represented in a 642-dimensional coordinate space, the 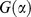 here is a one-dimensional function of the reduced curve parameter *α* only. Unlike the multidimensional free energy in the conventional string method [21], [24] as a function of all the coarse coordinates, here the 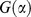 effectively integrates all degrees of freedom orthogonal to the curve, and properly incorporates factors such as the cross section of the transition tube [26]. Recent studies [27] demonstrated that such one-dimensional free energies are less sensitive to the choice of the representative (coarse) coordinates, and more faithfully characterize the transition than the high-dimensional free energies do.

Methods have been recently proposed to calculate the one-dimensional free energy profiles in a multidimensional conformational space. From confined simulations in Voronoi cells, e.g., the free energy can be obtained from the frequencies of the collisions at the cell boundaries [26], [27]. Here we adopted a new approach that generalizes the 1D umbrella sampling to compute the free energy profile along a curve. By invoking a local linear approximation, the biasing potential in each umbrella window acts only along the tangent direction of the curve, with all other directions in the conformational space unrestrained. The approximation is valid if the curve is sufficiently smooth such that its tangent direction only changes slightly over the distance between neighboring windows. The umbrella sampling can be combined with Hamiltonian replica exchange [38], as adopted in this study, to enhance the efficiency. The method presented here for the calculation of 1D conformational free energies can be conveniently implemented, and should be generally applicable to other systems. In the meantime it would also be desired to validate the method on simpler systems with clearer conclusions to compare.

Our calculated free energy profile indicates that without the bound ligand, the closed conformation of AdK is not metastable, which is also consistent with our unrestrained simulations here. By the end of all unrestrained simulations, only one (C8) did not approach the open state. Even in this simulation (C8), the protein still deviated from the crystal structure by some amount. We note that a single free energy minimum near the open state and an unfavorable closed conformation were also recently reported by Matsunaga et al. for the ligand-free AdK [18], and are consistent with previous simulation studies [13], [17] as well. The ∼13 kcal/mol free energy obtained here for the closed state is similar to the value of ∼20 k_B_T (∼12 kcal/mol) from the string-method calculation by Matsunaga et al. [18], although other simulations using different order parameters reported a wide range of values for this free energy difference in the ligand-free AdK. We note that because the closed state is not near a local minimum, its exact position along the order parameter might be somewhat ambiguous, which may give rise to some variation in the assigned free energy value.

Employing single-molecule FRET technique, Hanson et al. monitored the distance between two dyes attached to the LID and CORE domains, respectively, of an AdK mutant [15]. Using advanced statistical analysis, it was concluded that for the ligand- free AdK, the closed state is metastable and in fact even more favorable than the open state [15]. This appears to disagree with most simulations (including our results here) and knowledge inferred from some other experiments. We note that a single distance may bear a high degeneracy of the conformational states, given the large conformational fluctuations observed in the simulations. Possible multiple conformations of AdK, as discussed below, might contribute to the distribution of the measured distance. In light of this, simulations that more closely mimic the particular experiment, such as ones with the attached dyes explicitly incorporated, would help to gain more insight into this discrepancy.

Indeed, our unrestrained simulations revealed a substantial degree of conformational flexibility in AdK. In particular, the eight simulations (C1–C8) initiated from the closed-state crystal structure evolved quite differently, with most simulations (C1–C5) completing the transition and reaching the open conformation, but one (C8) staying near the closed state during the entire 200 ns. Scenarios from previous unrestrained simulations, such as complete closed-to-open transitions [22] and partial transitions [13] of the ligand-free AdK, were all observed in our multiple simulations here, suggesting that the variation is due to the intrinsic diversity of the conformational dynamics, rather than different simulation protocols. These simulations also suggest that some metastable states should exist between the open and closed conformations, which, however, are not captured by our free energy profile. The timing and pathway of the observed closed-to-open transitions are also not identical in different simulations (Fig. 3). Given such diversities, it appears that a single transition tube is not sufficient to accurately describe all the conformational states, and two or more transition pathways should be explored. The transition pathway adopted here may also have missed some off-track states which may trap the protein for substantial amounts of time. As discussed in Results, the large number of charged residues and some temporarily formed salt bridges may contribute to a rugged conformational landscape featuring multiple intermediate states.

Despite the large variation in the conformational dynamics here, our unrestrained simulations and umbrella-sampling simulations consistently indicate that the closed-state crystal structure is highly unfavorable for the ligand-free AdK. In contrast, conformational transitions of AdK with a bound ATP analog were characterized in earlier simulations [17], [18]. In particular, a recent study [18] revealed that both the open and the closed states of the ligand-bound AdK correspond to a free energy minimum, with the closed state energetically favored by ∼5 kcal/mol, in contrast to the ligand-free case with a higher free energy (by ∼12 kcal/mol) for the closed state, as similarly found here. These results thus suggest that a ligand-bound open state would be energetically more accessible than a ligand-free closed state, and further imply that ligand binding would precede the closing transition of AdK and reversely, ligand dissociation would follow the opening transition. Calculation of the ligand binding constants in the open and closed AdK conformations may shed light on this issue.
